# Exosomes in Cardiovascular Diseases

**DOI:** 10.3390/diagnostics10110943

**Published:** 2020-11-12

**Authors:** Marta Zarà, Patrizia Amadio, Jeness Campodonico, Leonardo Sandrini, Silvia S. Barbieri

**Affiliations:** 1 Unit of Brain-Heart axis: Cellular and Molecular Mechanisms, Centro Cardiologico Monzino IRCCS, via Parea 4, 20138 Milan, Italy; patrizia.amadio@ccfm.it (P.A.); leonardo.sandrini@ccfm.it (L.S.); 2 Intensive Cardiac Care Unit, Centro Cardiologico Monzino IRCCS, via Parea 4, 20138 Milan, Italy; jeness.campodonico@ccfm.it

**Keywords:** exosomes, diagnostic, biomarkers, cardiovascular disease, extracellular vesicles

## Abstract

Exosomes are nano-sized biovesicles of endocytic origin physiologically released by nearly all cell types into surrounding body fluids. They carry cell-specific cargos of protein, lipids, and genetic materials and can be selectively taken up by neighboring or distant cells. Since the intrinsic properties of exosomes are strictly influenced by the state of the parental cell and by the cellular microenvironment, the analysis of exosome origin and content, and their cell-targeting specificity, make them attractive as possible diagnostic and prognostic biomarkers. While the possible role of exosomes as messengers and a regenerative tool in cardiovascular diseases (CVDs) is actively investigated, the evidence about their usefulness as biomarkers is still limited and incomplete. Further complications are due to the lack of consensus regarding the most appropriate approach for exosome isolation and characterization, both important issues for their effective clinical translation. As a consequence, in this review, we will discuss the few information currently accessible about the diagnostic/prognostic potential of exosomes in CVDs and on the methodologies available for exosome isolation, analysis, and characterization.

## 1. Introduction

Since their discovery in the early 1980s [1,2], it has become clear that exosomes are essential mediators of cell-to-cell communication and contribute to many physiological and pathological aspects [3]. Exosomes are nanovesicles of endocytic origin and they are secreted by nearly all cell types as part of their normal physiology. However, the intrinsic properties of exosomes are strictly influenced by the state of the parental cell and by the cellular microenvironment. Their specific way of formation makes them faithful messengers that carry information about the original cellular/tissue context in the form of cell-specific surface markers and/or membrane-enclosed biomolecules. Moreover, exosomes protect their cargo from clearance or damage thanks to their double-layered membrane, thus prolonging their circulation half-life. Consequently, analysis of exosome origin and content, together with their availability in all body fluids, make them attractive as possible clinical biomarkers providing information about the nature, severity, and prognosis of a particular disorder, thus improving, with other biomarker currently used, the disease diagnosis and prognosis.

While the potential of exosomes as biomarkers is becoming important in several clinical settings [4,5,6,7,8,9,10,11,12,13,14,15,16], effective and selective isolation of exosomes and the characterization of their cargo has turned out to be a major challenge in adopting exosomes as a source of biomarkers in clinical practice. Indeed, the process of exosome isolation as well as the methodologies for their characterization are still undergoing optimization [17].

The possible role of exosomes in cardiovascular diseases (CVDs), particularly in ischemic heart disease, as messengers or as a regenerative tool is actively investigated by several groups [18,19,20,21,22,23], but the evidence about their usefulness as a diagnostic and prognostic tool is still limited mainly due to the lack of consensus regarding the most appropriate approaches for exosome isolation and characterization.

In this review, we will critically discuss the methodologies currently available for exosome isolation and analysis of their cargo since they represent the most challenging issues for the clinical transition of exosomes, including in the cardiovascular field. In addition, we will present recent evidence about the role of exosomes in CVDs, focusing on their diagnostic and prognostic potential.

## 2. Exosomes Biogenesis

**Figure 1:**
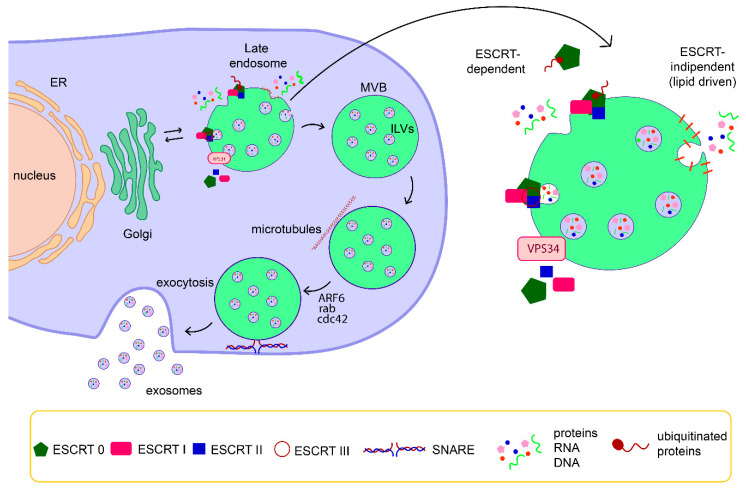
Schematic representation of exosome biogenesis and release. Exosomes are generated by invagination of late endosomal membranes that results in the formation of intraluminal vesicles (ILVs) within multivesicular bodies (MVBs). The formation of ILVs is driven by endosomal sorting complex required for transport (ESCRT) components and /or by specific membrane domains rich in lipids. Then, MVBs travel along microtubules and ILVs are ultimately secreted as exosomes through a SNARE-mediated fusion of MVBs with a plasma membrane.

Exosomes are generated by invagination of late endosomal membranes that results in the formation of intraluminal vesicles (ILVs) within multivesicular bodies (MVBs) (Figure 1). Upon exocytosis of MVBs, ILVs are ultimately secreted as exosomes with a size range of ~30 to 160 nm in diameter [24].

The endosomal sorting complex required for transport (ESCRT) is a key mediator of MVB biogenesis. The ESCRT is a complex machinery composed of four separate proteins (0 through III) that works cooperatively to facilitate MVB formation, vesicle budding, and protein cargo sorting [25,26]. The ESCRT mechanism is initiated by recognition and sequestration of ubiquitinated proteins to specific domains of the endosomal membrane via ubiquitin-binding subunits of ESCRT-0. After interaction with the ESCRT-I and -II complexes, the total complex then combines with ESCRT-III, a protein complex that is involved in promoting the budding processes. Finally, after cleaving the buds to form ILVs, the ESCRT-III complex separates from the MVB membrane with the energy supplied by the sorting protein Vps4 [25]. Despite the controversy on the release mechanism of exosomes mediated by ESCRT, different ESCRT components and ubiquitinated proteins have already been identified in exosomes isolated from various cell types. Additionally, the typical exosomal protein Alix, which is associated with several ESCRT (TSG101 and CHMP4) proteins, has been reported to participate in endosomal membrane budding and abscission, as well as exosomal cargo selection via interaction with Syndecan [27,28]. Nonetheless, it has been also suggested that vesicle formation and subsets of ILVs can be generated by an ESCRT-independent mechanism, through a lipid-driven fashion [24,29,30,31]. Proteins, such as tetraspanins, also participate in exosome biogenesis and protein loading. Tetraspanin-enriched microdomains (TEMs) are ubiquitous specialized membrane platforms for compartmentalization of receptors and signaling proteins in the plasma membrane [32]. The vesicle pinching off in the lumen of MVB is sustained by the polymerization of actin cytoskeleton, which in turn is regulated by several small GTPases, including ARF6, Cdc42, and Rab-family members. The last step of exosomes biogenesis, the exocytosis of ILVs, is still poorly understood, but a few molecular players involved in this process have been identified, including small GTPases of the Rab family (Rab11, Rab27, and Rab35) and the SNARE complex [24,33].

It is likely that the mechanisms of exosomes biogenesis and release in the context of CVDs are similar to those above described. Nevertheless, as already said, exosome generation and release are highly sensitive to several factors and presumably also to cardiovascular system alteration. Clear examples of this aspect are provided by works on cultured cardiomyocytes showing an alteration of exosome generation and release upon glucose deprivation [34] or treatment with TGF-β and PDGF [35]. Moreover, stress conditions, such as hypoxia, inflammation, or injury, induce an increase in the release of exosomes from cardiomyocytes as well as an alteration in exosome cargo [36,37,38]. In addition to in vitro and animal models, human studies actually demonstrate that alteration in release of exosomes and in their cargo has been found in CVDs patients [39,40,41,42].

## 3. Exosome Isolation

The isolation of pure exosomes is a critical step to understand their physio-pathological role and for their clinical application in several diseases, including CVDs. However, exosome isolation is challenging and there are no currently reliable protocols able to isolate with absolute precision exosomes from other extracellular vesicles in a given biological specimen. Especially when working with biological fluids, the enrichment/purification of exosomes from a given sample is necessary to enhance the exosome signal over non-exosomal noise; however, each methodology currently used for exosome purification likely exerts distinct bias contingent on the sample used and on possible contaminants co-purified with the exosomes. Moreover, the method used for exosomes isolation highly impacts on the quality of exosomes isolated and thus on the downstream analyses. Hence, the implementation of the purification techniques is a fundamental prerequisite to obtain reliable data in exosome research. The most commonly used techniques for exosomes isolation, as well as their advantages and disadvantages, are summarized in Table 1. In addition, some examples of their application in the study of exosomes in the cardiovascular field are reported.

### 3.1. Ultracentrifugation

**Figure 2:**
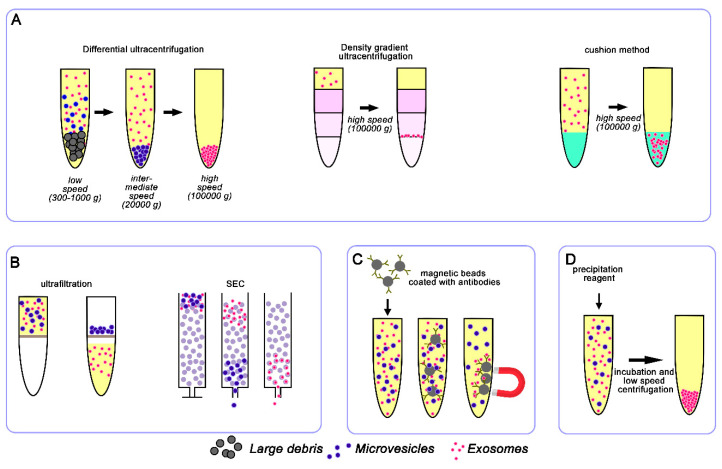
Schematic representation of conventional exosome isolation techniques. The most widely used techniques are based on ultracentrifugation (**A**), size (**B**), immunoaffinity (**C**), and precipitation reagents (**D**).

Differential ultracentrifugation is currently the most commonly used method for exosomes isolation. It usually consists of a series of centrifugation cycles of different centrifugal force and duration to separate exosomes from other sample components (Figure 2). The different centrifugation steps are performed with increasing centrifugal force in order to get rid of cells, cell debris, and larger extracellular vesicles (like microvesicles) that otherwise might contaminate the final exosome population. In order to pellet exosomes, the centrifugal force of the last step typically ranges from ~100,000 to 120,000× *g*. Finally, the isolated exosomes are resuspended in the appropriate medium and used for downstream analyses. Despite the wide use of ultracentrifugation for exosome isolation, it requires costly instrumentation and is time-consuming. Moreover, ultracentrifugation is also not always applicable to clinical samples due to the required volume of starting material and it is not able to efficiently separate exosomes from other co-isolated biomolecules (e.g., lipoproteins, protein aggregates). In addition, repeated “washing” ultracentrifugation steps can reduce the amount of co-isolated contaminants but they can damage the vesicles and reduce their yield [62,63].

Variants of ultracentrifugation also exist, such as density gradient ultracentrifugation that exploits differences in vesicle size and density through the creation of a discontinuous density gradient in a centrifuge tube with progressively decreased density from the bottom to the top (Figure 2). The sample is usually layered onto the top of the density gradient medium (typically sucrose and iodixanol) and subjected to an extended round of ultracentrifugation. During this step, the vesicles in the sample travel through the gradient until they reach the point at which their density matches the one of the surrounding solution. The separated exosomes are then conveniently recovered by simple fraction collection and can be further purified by ultracentrifugation or other techniques, such as size exclusion chromatography [64].

Frequently, exosomes isolated by ultracentrifugation are further purified through an extra purification step using a sucrose cushion to increase purity by removing protein contamination [65]. The density of sucrose (1.12 to 1.18 g/mL) is similar to that of exosomes (1.15 to 1.19 g/mL) and hence produces a cushioning effect, maintaining integrity of exosomes and separating protein contaminants with higher density (1.22 g/mL). Although the purity of the sample is quite high, the yield is very low with this 2 step-method. To limit vesicle loss and damage, Gupta and colleagues proposed a one-step sucrose cushion ultracentrifugation method [66], in which conditioned media containing exosomes is directly loaded on 30% sucrose gradient and centrifuged at 100,000× *g*, 4 °C for 90 min (Figure 2). The supernatant is discarded and the sucrose layer resuspended and ultracentrifuged at 100,000× *g* for 90 min to pellet down the exosomes. The exosomes isolated by this method maintain integrity and the recovery yield is higher compared to ultracentrifugation alone [66].

The use of cushion method combined with density gradient ultracentrifugation has been used too [67]. In this methodology, exosomes are firstly concentrated by using 60% iodixanol cushion to maximize exosome recovery and for a better preservation of their physical and biological properties. Then, the concentrated exosomes are separated through density gradient ultracentrifugation to remove non-exosome nanoparticles and protein contaminates. This method is time-consuming but allows for high purity exosomes and, importantly, the biological inertness of iodixanol is suitable for downstream functional assays.

### 3.2. Size-Based Isolation Methods

Size-based isolation methods merely depend on size or molecular weight. Exosome separation based on their size can be achieved by differential passages through physical barriers, using filters or chromatography columns (Figure 2).

As in conventional filtration, isolation method exploiting ultrafiltration relies on the size or molecular weight. This technique employs membranes with pores of different diameters and/or molecular-weight cut-off membranes to isolate exosomes [68]. Ultrafiltration is rapid and does not require expensive equipment but, as with ultracentrifugation, it does not allow the removal of contaminating proteins. Filtration methods are often combined with ultracentrifugation, where physical membranes are used as the first cleaning step to sieve cells and larger vesicles [69].

Size exclusion chromatography (SEC) is an additional size-based separation technique applied to exosome isolation that uses a stationary phase consisting of resin particles with known porous size.

Larger particles are excluded from entering the pores and thus are eluted from the column earlier. Molecules and small particles are longer retained into the pores of the stationary phase and elute later. Similarly to density gradient centrifugation, SEC has been shown to allow reduction of contaminant proteins in the exosome population [70,71,72,73]. SEC separates plasma exosomes from high density lipoproteins (18–23 nm) [73], but fractions isolated can still contain a small amount of lipoproteins such as chylomicrons (100–600 nm) and very low density lipoproteins [VLDL (30–80 nm)] [73,74,75]. This method has been successfully used for small scale analysis of exosomes from clinical samples [72,76]. Nevertheless, as for the other techniques, SEC has several technical and practical limitations. First, SEC only allows efficient isolation of exosomes larger than the pore size of the matrix of the stationary phase used (i.e., 70 nm for CL-2B Sepharose), thus excluding the smaller vesicles. Moreover, the vesicle yield is generally low, the purified sample is diluted and may require an additional concentrating step [72]. Despite the shorter processing time compared to differential ultracentrifugation, SEC still requires significant hands-on time for column preparation, washing, and (re)equilibration. In addition, manual collection of fractions may introduce operator-dependent variability. However, these last limitations are overcome by recently developed commercial systems that provide both columns and an automatic fraction collector for fast and automated isolation of exosomes.

### 3.3. Immuno-Affinity Purification

Exosome membranes are known to contain large quantities of proteins and thus immune-affinity capture may be used to isolate them, exploiting the interactions between these proteins (antigens) and specific antibodies [77] (Figure 2). Immuno-affinity purification methods selectively capture specific exosomes from a complex population based on certain surface markers. Generally, this approach employs magnetic beads covalently coated with streptavidin, which can be coupled in a high-affinity fashion to any biotinylated capture antibody. This method provides promising results for the isolation of subgroups of exosomes derived from a specific cell type [78]. The immune-affinity method is compatible with routine laboratory equipment but requires multiple steps in sample preparation, making the isolation process prone to errors.

### 3.4. Polymer-Based Precipitation

Precipitation methods represent an easy and fast approach for exosome isolation which is mostly exploited by commercial kits and is becoming largely employed with clinical samples. Precipitation-based methods work by altering the solubility of exosomes, thus allowing their isolation from the solution (Figure 2). For this purpose, the sample is mixed with water-excluding polymers, such as polyethylene glycol (PEG), that tie up water molecules and force less soluble components out of solution. Generally, the biological fluid is incubated with a precipitation solution and, after incubation, the precipitate containing exosomes is isolated by low speed centrifugation. This isolation method is easy, does not require any specialized equipment, has a high recovery rate, is scalable for large sample sizes, and allows easy integration into clinical usage. Despite the high recovery yield, exosomes isolated with precipitation reagent usually contain a lot of contaminating proteins [70,79]. Nevertheless, some commercially available kits are implemented to reduce the contamination with other proteins, like albumin or immunoglobulins. Another problem of this methodology is that the polymer present in the exosome sample may interfere with the downstream analyses.

### 3.5. Microfluidics-Based Isolation Techniques

New techniques are essential to meet the challenge of providing high-purity exosomes for clinical practice. Recently, microfluidics-based technologies have become important techniques for the microscale isolation and detection of exosomes. This technique exploits both physical and biochemical properties of exosomes together with innovative sorting mechanisms, such as acoustic, electrophoretic, and electromagnetic manipulations [80,81]. The approaches employed by these microfluidic platforms are wide, including immunoaffinity, membrane-based filtration, trapping on nanowires, acoustic nanofiltration, deterministic lateral displacement (DLD), and viscoelastic flow sorting. Microfluidic platforms, taking advantage of immunoaffinity capture, are the most commonly used and, in these platforms, the antibodies targeting exosomal markers are immobilized on solid surfaces. Based on the surface functionalized for exosome capture, platforms can be divided in devices with inner surface(s) modified and devices that employ capture beads. Microfluidic devices with inner capture surface(s) are specifically designed to enhance the interaction between targeted exosomes and the functionalized surface(s). For instance, Chen and colleagues, in their pioneering work [82], used a microfluidic immunoaffinity platform exploiting herringbone groves to increase the capture efficiency in a straight flow surface-modified channel [82]. Another example of immuno-affinity-based approach is the “ExoChip” proposed by Kanwar and colleagues [83]. In this microfluidic platform, multiple circular capture chambers are interconnected by narrow-channels that increase exosomes retention time, while straight channels present in between these circular areas allow intermittent mixing. Furthermore, the ExoChip is geometrically designed so it can be read by a standard plate reader [83]. In devices employing functionalized capture beads, microfluidic devices exploit sample-bead interactions and subsequent separation of the beads. In the simplest case, the sample is incubated with capture beads off-chip, and only downstream bead separation step takes place on-chip.

Other devices use the microfluidics-based membrane filtration approach isolating exosomes by their size. The first example of such device is a nanoporous membrane with an adjustable pore size that has been inserted in a microfluidic chip, and the authors developed two kinds of devices exploiting pressure- or electrophoresis-driven filtration to separate EVs [84]. In addition, a multiscale filtration device based on the nanowire traps system was developed by Wang and colleagues [85]. They built a ciliated nanowire-on-micropillar structure that traps specifically sized liposomes that are eventually released by dissolving the porous silicon nanowires in PBS buffer [85]. Finally, a type of microfluidic device based on pillar-array that can sort particles in a continuous flow through nano-deterministic lateral displacement [86] has been proposed by Huang and colleagues. They successfully developed a microfluidic device that is based on the asymmetric bifurcation of laminar flow around obstacles. In this device, the particles take the path deterministically on the basis of their size and the separation process uses laminar flow through a periodic array of micrometer-scale obstacles [87]. Even though these systems seem to be very promising, they still need more implementations.

## 4. Exosome Characterization

The accurate determination of exosome characteristics is required to determine their biological meaning but also their feasibility as a potential biomarker in the clinical routine. So far, several techniques were employed for exosome characterization but, as for isolation methodologies, their use is still under debate and each of these techniques has its own limitations that must be taken into consideration. At the present, the potential use of exosome as a biomarker of diseases focused on exploring their concentration and specific protein/miRNA cargo [4,5,7,8], and only few studies investigated the relationship between exosome dimension and pathological conditions [39,88]. The techniques most commonly used for exosome characterization, including their application in the cardiovascular field, are summarized in Table 2.

### 4.1. Nanoparticle Tracking Analysis

Nanoparticle tracking analysis (NTA), first applied to exosome characterization in 2011, is a light-scattering technique that allows sizing and enumeration of nanoparticles in liquid suspension [91]. NTA, using the tracking of the Brownian motion of nanoparticles in a liquid suspension, measures the concentration and size distribution of particles in 0.01–1 µm diameter range. The particles in suspension are detected by incident laser light while a light-sensitive CCD camera records the scattered light of the particles and, finally, the software tracks the motion of vesicles and calculates their diameter based on the Stokes–Einstein relationship [92]. The outputs of this method are particle size distribution and concentration. The use of the NTA in exosomes analysis has several advantages. In addition to an easy sample preparation and fast analysis/measurements, NTA, having high resolution, measures accurately nanovesicles and, importantly, sample acquisition is performed in a liquid phase limiting alterations to the studied vesicles [92]. Alongside advantages, the NTA technique has also some limitations. First of all, there are important technical cautions for the appropriate use and the reproducibility of the measures. A critical parameter is the proper dilution of the sample: to obtain a reliable measurement, the “right” dilution factor should be used. The NTA camera has to register all the vesicles present in a sample and the overlaying effect of larger vesicles, masking smaller ones should be avoided [92,93]. The second NTA limitation concerns the detection of a fluorescent signal. Although the NTA system is equipped with a fluorescent module, its real application is still limited. Indeed, the fluorescent signal must be very bright to be detected by the NTA system [94], and exosomes phenotyping using directly labeled fluorescent antibodies does not give good results, unless the expression of the studied marker is high [94]. Nevertheless, exosomes labeling with fluorescent dyes incorporated into the membrane seems to be adequate for particle detection. Finally, NTA failed to distinguish exosomes from protein aggregates, liposomes, and other nano-contaminants.

### 4.2. Dynamic Light Scattering

Dynamic light scattering (DLS) is an alternative technique for measuring the dimension of exosomes. In this technique, a monochromatic and coherent laser beam passes through a suspension of particles and, when it encounters a particle, the laser light is scattered in all directions [95]. Particle size is determined from fluctuations in scattered light intensity due to their Brownian movement but, differently from NTA, the particles are not visualized. DLS is easy to use but the system provides reliable data only when working with a monodispersed suspension, whereas results related to suspensions of particles ranging in different sizes are strongly influenced by the contribution of larger particles even if they are present in a low quantity [95,96].

### 4.3. Resistive Pulse Sensing

Tunable resistive pulse sensing (TRPS) measures physical properties of exosomes, such as absolute size, concentration, and surface charge (ζ potential) [97,98]. These characteristics are determined by the electrical resistance of the particles passing through a size tunable nanopore filled with an electrolyte. Passing through the pore, the particle resistance increases, generating a pulse that is directly proportional to the particle volume. The main disadvantages of this technique are linked to system stability and sensitivity. Indeed, from one hand, the pores may be easily blocked by particles and, on the other hand, they may be too small to generate a signal higher than the background noise of the system [99].

### 4.4. Electron Microscopy

Transmission electron microscopy (TEM) is currently the most used imaging methodology to gain information about exosome morphological structure. The working principle of TEM is the creation of images generated from a beam of electrons passing through a sample, where a secondary electron is generated. These electrons are collected and magnified using special lenses. The great advantage of TEM is its high resolution power that allows to discriminate vesicles from other similar-size non vesicles. Nevertheless, sample preparation for TEM requires dehydration, chemical fixation, or staining of samples that may induce change in the morphology of the exosomes. Moreover, in some cases, the electron beam may also damage biological samples. Through TEM it is possible to label specific exosome antigens using immunogold staining, and TEM images can also be used to determine particle size.

Cryo-electron microscopy (cryo-EM) is a variant of electron microscopy where the samples are analyzed at cryogenic temperature. This EM technique presents major advantages over conventional TEM and is considered the best method for visualizing nanoparticles without dehydration and fixation artifacts [100]. Moreover, cryo-EM has an improved overall resolution when compared to TEM that allows to capture images of exosome lipid bilayer. Despite these advantages over the traditional TEM, cryo-EM is not widely applied due to instrument availability and time needed for sample analysis.

### 4.5. Atomic Force Microscopy

Atomic force microscopy (AFM) represents a unique alternative to electron diffraction techniques for studying exosomes [101]. By detecting the interactions between a probing tip and the sample surface, this technique provides information about abundance, structure, biomechanics, and biomolecular content of individual exosomes. An important feature of AFM is that the sample is analyzed in native conditions, with minimal sample preparation and without any destructive mode of operation [102,103]. The disadvantage is that analysis may be influenced by the different experimental conditions, like temperature, state of the AFM tip, force between probe and sample, or varying scan speed. Different AFM imaging modes have been successfully applied to exosomes analysis, like contact mode, tapping mode, non-contact mode, peak force tapping, and single molecule-force spectroscopy [101].

### 4.6. Flow Cytometry

Over the last two decades, flow cytometry has been regarded as one of the most commonly used techniques for EVs analysis [104]. Although the new generation of flow cytometers use multiple angles for forward scatter detection, which provides an improved particle resolution [105,106,107], exosomes fall below the resolution of flow cytometry. Suarez and colleagues used the bead-assisted method for the semi-quantitative analysis of exosomes [108], mixing exosomes with aldehyde/sulfate-latex beads, and then incubating bead-coupled exosomes with antibodies prior to analysis.

### 4.7. Raman Spectroscopy

Raman spectroscopy is a vibrational technique based on Raman scattering that is becoming popular in many areas, including biology and medicine. In this methodology, the sample interacts with incoming monochromatic laser light and the photons change their energy by exciting vibrational modes of molecules. This vibrational technique measures the inelastically scattered photons, resulting in a Raman spectrum that corresponds with known vibrations of specific chemical groups [109]. This method allows to identify the molecular composition of a sample with very high accuracy, to provide information on the chemical structure of exosomes [110], and to differentiate exosomes on the basis of the membrane lipid/protein content along with other various surface modifications. Of great importance is that Raman spectroscopy is a label-free and a non-destructive technique. Finally, the Raman signal can be enhanced by employing noble metal nanoparticles allowing the use of a small amount of exosomes for the detection and analysis.

### 4.8. Exoview Platform

Recently, NanoView Bioscience has developed the ExoView R100 platform which is an affinity-based technology specifically dedicated to exosome cargo analysis. Briefly, exosomes are immobilized on ExoView chips by affinity capture usually against exosomal transmembrane proteins. Once captured, exosomal cargo can be studied permeabilizing the sample with the ExoView Cargo Kit to enable access of antibodies. This platform allows specific populations of exosomes to bind in a multiplexed manner to a functional chip and to measure both internal and external proteins, as well as to perform co-localization studies on single exosome. In addition, exosome subpopulations can be quantified in terms of vesicle size and number. The sample volume required is small and it must be underlined that purification biases are reduced, since the sample can be directly read without the preliminary step of vesicle isolations.

### 4.9. Other Techniques

Most of the current available works characterize exosome by widely used approaches. Specifically, the protein content is usually quantified by common assays (BCA, Bradford assays) and the expression of specific proteins and/or exosomal markers is usually investigated by Western blot analysis or by enzyme-linked immunosorbent assay (ELISA). Finally, omics approaches, including proteomic analysis as recently described by Mallia and colleagues [111], are frequently used to explore exosomal cargo for identification of new potential biomarkers. Like exosomal proteins, exosomal miRNAs have been largely investigated in several pathological contexts in an effort to gain a better understanding of exosomes and to investigate whether they can be used as potential biomarkers for diagnosis [79,112,113].

## 5. Clinical and Biomedical Values of Exosomes in CVDs

Despite the progress in prevention and treatment of CVDs, this pathology still represents the most common cause of death and comorbidity in industrialized countries. Thus, further exploration of the pathophysiology of CVDs is important to provide new targets of disease, as well as novel biomarkers and/or therapeutic approaches. Increasing evidence in the field of CDVs strongly suggests a role for exosome-mediated intercellular communication in maintaining the homeostasis of the cardiovascular system, thus representing a potential therapeutic target [19,114,115,116,117]. However, the clinical/biomedical interest towards exosome research in the cardiovascular field is mainly due to the promising results in cardiac regeneration as well as in disease diagnosis/prognosis.

### 5.1. Therapeutic Potential of Exosomes in CVDs

In CVDs context, growing evidence suggests a role for these nanovesicles in cardioprotection and, thus, their potential use as a therapeutic tool. In myocardial infarction, reperfusion of the occluded coronary vessel leads to (ischemia-)reperfusion injury (I/R injury) and reduction of this detrimental effect is crucial to improve the survival of AMI patients. Interestingly, several studies demonstrated that the effect of remote ischemic conditioning (RIC), a strategy to protect the heart against I/R injury, is strongly related to the recruitment of circulating exosomes [118,119,120]. It has been recently shown that exosomes play a critical role in the efficacy of RIC not only in the acute phase of MI, but also in the chronic phase of MI, preventing left ventricular (LV) dysfunction [43]. Indeed, Yamaguchi and colleagues, using an animal model of chronic heart failure, demonstrated that RIC treatment induced recruitment of the exosome rich in miR-29a and insulin-like growth factor 1 receptor (IGF-1R), both biomolecules with protective roles against cardiac fibrosis and remodeling [43]. The therapeutic potential of exosomes is also showed by other authors in different experimental settings. For example, exosomes released by cardiac progenitor cells (CPCs) display angiogenic effects [44], reduce myocyte cell death in an animal model of AMI [54], improve cardiac function and reduce fibrosis in a rat model of IR injury [121]. One of the most interesting aspects related to exosomes cardioprotection is that, in the last years, several works have demonstrated that the beneficial effects of stem cells on cardiac function after AMI are actually mediated by exosomes. After the first results on the effects of exosomes released from mesenchymal stem cells (MSCs) [48,51,122], several groups explored the functional consequences on cardiac function of exosomes released by MSCs, cardiac stem cells (CSCs), embryonic SCs, hematopoietic SCs, cardiosphere-derived SCs, and plasma. Actually, mouse embryonic SC-derived exosomes enhance neovascularization, cardiomyocytes, and CPC survival, and reduce post-infarction fibrosis [123]. Similarly, exosomes released from cardiosphere-derived cells in a porcine model of acute and chronic myocardial infarction reduce scar, fibrosis, prevent hypertrophy, and increase cardiomyocytes proliferation [55]. Despite the efficacy of exosomes derived from stem cells in cardioprotection and regeneration, one of the main challenges is their delivery to the heart. The intravenously administration has been proved to be a not optimal method for exosome cardiac delivery, since the majority of exosomes injected are trapped in the liver [124]. By contrast, the invasive procedure of intramyocardial exosome injection has led to better and more effective results compared to coronary injection [55]. To overcome these limitations, Vandergriff and colleagues showed the advantages of creating infarct-targeting exosomes, through the use of cardiac homing peptide, to increase the efficacy of intravenously delivered exosomes [46].

### 5.2. Diagnostic Potential of Exosomes in Cardiovascular Diseases

In the last years, the scientific interest toward exosomes is enormously increased for their possible implication in clinical application. The number, origin, and cargo of circulating exosomes differ under pathological conditions, suggesting their potential as a biomarker of disease. Although evidence about the importance of exosomes in cardiovascular disease is continuously expanding, their clinical application as markers for a specific disease is still strongly limited especially by the technological difficulties. Indeed, most of the current studies are performed in vitro and/or in animal models, whereas works about exosomes characteristics in cardiovascular patients are still scarce.

#### 5.2.1. Exosomes in Coronary Artery Disease

Coronary artery disease (CAD) is the most common type of heart disease and it represents the leading cause of global mortality. Despite advances in treatment and prevention strategies, patients with established CAD still face a high residual cardiovascular risk, demonstrating the great need of novel, reliable tools for risk stratification to guide secondary preventive therapies. The principal cause of CAD is atherosclerosis, a chronic pathological condition characterized by the accumulation of lipids and fibrous elements in the large arteries. The mechanism underlying atherosclerosis is complex and involves multifactorial causes among which oxidative stress, endothelial dysfunction, and inflammation are considered the most important. Exosomes may contribute to atherosclerosis development and progression by means of their role as intercellular messengers. For instance, exosomes released by endothelial cells (ECs), upon activation of CD137 inflammatory signaling, promote plaque formation by inducing proliferative and migratory phenotype in vascular smooth muscle cells (VSMCs) as well as intimal hyperplasia after arterial injury [125]. Exosomes derived from nicotine-stimulated macrophages can predispose to atherosclerosis inducing VSMCs migration/proliferation through a mechanism PTEN-related [126]. Similarly, exosomes derived from foam cells transferring integrins to VSMCs, promote their migration and activate downstream signaling pathways [127]. In addition, exosomes isolated from serum of atherosclerotic patients, as well as exosome released by ECs exposed to oxidized low-density lipoprotein (ox-LDL), induce neutrophil extracellular traps (NETs) through the transfer of the long non coding RNA (lncRNA) MALAT1. Moreover, exosomes derived from ox-LDL-treated ECs exaggerate atherosclerosis development in mouse model of the disease [128]. The importance of another exosomal lncRNA (GAS5) in atherosclerosis progression has recently been provided by Chen and colleagues, demonstrating that exosomes carrying lncRNA GAS5 enhanced apoptosis in vascular endothelial cells [129].

As above mentioned, only few studies explore the potential diagnostic implication of exosomes in CAD and in its acute clinical manifestation, the acute coronary syndromes (ACS). All these works are mainly focused on the identification of specific miRNA or lncRNAs in exosomes, that have been demonstrated to play a critical role in cardiovascular development and disease and that may be potential markers of CAD [130,131]. Indeed, it has been demonstrated not only that exosomes contain RNAs [132], but also that exosomes include and protect circulating micro RNA (miRNAs) and lncRNAs from degradation, making them relatively stable in blood [133].

At this regards, Wang and colleagues analyzed the exosomal expression of miRNAs previously related to the pathogenesis of CAD and they detected a higher expression of miR-30e and miR-2a in plasma exosomes of patients with coronary atherosclerosis compared to healthy controls [41]. Similarly, greater amount of exosomal lncRNA HIF1A-AS1 was found in patients with atherosclerosis compared with healthy subjects [47].

Several exosomal miRNAs have been also related to ACS. In particular, miRNA-208a expression is significantly higher in the serum exosomes of ACS patients compared to healthy controls, and ACS patients with higher expression of miRNA-208a have a reduced survival rate during one-year follow-up. Interestingly, a positive association between serum exosomal miRNA-208a expression and Killip class, troponin peak, and LDL has been found in the ACS group [56]. This miRNA has been proposed to have a higher sensitivity compared to troponin, since its elevation becomes detectable in AMI patients before troponin [134]. Emanueli and colleagues provided evidence that also plasma exosomes well reflect the pathological condition. They showed that concentration of plasma exosomes increased in patients undergoing coronary artery bypass surgery for up to 48 h after surgery, as well as the expression of exosomal miR-1, miR-24, miR-133a, and miR-133b [40]. In addition, exosome concentration as well as the exosomal levels of miR-1, miR-133a, miR-24, miR-210, and miR-133b positively correlate with troponin, whereas their plasma levels do not [40], strongly suggesting the potential of exosomes as a marker of disease. Finally, the exosomal miR-83 is markedly upregulated in patients with myocardial infarction compared with healthy individuals and its expression increases with the degree of myocardial ischemic injury [49]. However, in the study, the authors do not indicate the time from the infarct event in which the analysis has been performed.

Another important aspect is that hypoxia has been demonstrated to alter exosome cargo. Indeed, cardiomyocytes under hypoxic conditions release exosomes carrying the cytokine TNFα [135] and the heat shock protein HSP60 [36]. Therefore, it is possible that in vivo, following acute myocardial infarction (AMI), a cardiomyocyte in a state of hypoxia, but not necrotic yet, might release signals through exosomes into the blood. On this basis, Kuwabara and colleagues suggested that the high levels of miRNA-133a detected in serum of ACS patients are included in exosomes released from suffering cardiomyocytes [136], hypothesizing their potential use as a biomarker of cardiomyocyte death.

This in vitro evidence paves the way to the use of exosome-associated proteins (and miRNAs) as “pre-necrotic” biomarker(s), a signal actively released by the injured heart before necrosis begins.

#### 5.2.2. Exosomes in Heart Failure

Many CADs progress up to chronic heart failure (HF), and the number of patients developing HF is increasing each year. Despite the significant advances in therapies and prevention, mortality and morbidity in patients suffering from HF are still high and quality of life is poor [137]. HF is a condition in which the heart cannot pump enough blood to meet the body’s needs. Among the factors contributing to HF development, adverse cardiac remodeling following myocardial infarction is one of the most important and exosomes have been demonstrated to have a role in this mechanism, suggesting their potential utility as an early biomarker [138]. In addition, several studies show that degeneration of endothelial function and vascular integrity, imbalanced angiogenesis, and inflammation critically contribute to the progression of HF.

Recently, Matsumoto and colleagues suggested that exosomes-bound miRNAs can be predictors of ischemic HF in post-AMI patients [57]. They showed that in exosomes isolated from sera of patients 18 days after AMI, the levels of p53-responsive miRNAs (miR-192, miR-194, and miRNA-34a) were markedly higher in those patients who would have experienced HF within 1 year from AMI onset. Moreover, the expression levels of exosome miR-194 and miR-34a, but not miR-192, positively correlated with left ventricular diastolic dimension and left ventricular ejection fraction measured ≈1 year after the onset of AMI. This finding suggests that exosome-associated p53-responsive miRNAs may predict left ventricular remodeling after the convalescent stage of AMI [57]. Similarly, HF patients display higher levels of miRNA-92 in serum exosomes [139], and greater amounts of miR-21 and lower levels of miR-425 and miR-744 in plasma exosomes compared to healthy control [58]. Of note, all these miRNAs are usually associated to fibrosis, suggesting their potential use as biomarkers in early diagnosis of hypertrophic heart diseases. Finally, it should also be taken into account that chronic HF patients have an higher concentration of plasma exosomes [42].

#### 5.2.3. Exosomes and Cerebrovascular Disease

Exosomes content may be used as a valuable biomarker also in cerebrovascular diseases. For instance, in patients with acute ischemic stroke, serum concentration of exosomes, as well as exosomal levels of brain-specific miRNAs (miR-9 and miR-124), increase compared to controls. Moreover, the level of miR-9 and miR-124 positively correlate with scores of the National Institutes of Health Stroke Scale (NIHSS), the infarct volumes and the IL-6 serum levels [59], suggesting the potential use of exosome also for evaluating the damage degree caused by ischemic injury. Chen and colleagues [60] reported that exosomal miR-223 levels were higher in acute ischemic stroke patients compared to the control group, and its level increased progressively until 72 h post stroke and positively correlated with NIHSS scores [60]. Of note, increased exosomal miR-223 was associated with acute ischemic stroke occurrence, stroke severity, and short-term outcomes [60]. Moreover, the serum exosomal neuronal synaptopodin is a promising biomarker in neonates affected by acute hypoxic–ischemic encephalopathy since its levels are associated with short-term neurologic outcomes [52].

#### 5.2.4. Exosomes in Other CVDs

The role played by exosomes in other CVDs, such as cardiac arrhythmia, valve diseases, cardiomyopathies, is poorly understood, and available data are very limited. However, there are recent in vitro and animal studies suggesting a potential role of exosomes in the pathophysiology of the above-mentioned diseases. Cardiomyocytes derived/isolated from CAD patients or from the ischemic rat model release exosomes rich in miR-1 and miR-133 [61,136], two miRNAs that can predispose to cardiac arrhythmia through the modulation of action potential and cardiac conduction via the Ca2 + /calmodulin-dependent protein kinase II signaling [140,141]. Other evidence suggests that exosomes can be also involved in the pathophysiology of diabetic cardiomyopathy (DCM) [142]. DCM is a severe complication in diabetic patients, accompanied by impaired left ventricular systolic or diastolic function, ventricular hypertrophy, interstitial fibrosis, and myocardial microvascular rarefaction. The exosomes released from cardiomyocytes of type 2 diabetic rats contain a greater amount of miR-320, able to reduce NO production and to inhibit angiogenesis in coronary endothelial cells [143]. In addition, a potential link between exosomes and septic cardiomyopathy has been recently proposed starting from the evidence that plasma exosomes isolated from shock patients induced vascular apoptosis and myocardial dysfunction [90]. The inhibition of exosome release decreases in vitro the production of pro-inflammatory cytokines and, in a septic mouse model, prevents the inflammatory response, mitigates the cardiac dysfunction, and improves the survival [144]. In addition, the iNOS present in exosomes can sustain NO production, previously related to myocardial dysfunction in sepsis [145,146].

In regard to valve diseases, the evidence of a possible role of exosomes is further limited. Mitral valve prolapse (MVP) is a common valvular heart disease that can lead to mitral regurgitation and subsequent congestive heart failure. Naturally occurring myxomatous mitral valve disease (MMVD) in dogs closely resembles MVP in humans and it has been associated with increased levels of plasma-associated exosomal cfa-miR-9, cfa-miR-181c, cfa-miR-495, and cfa-miR-599. Interestingly, in this canine model of disease, the expression levels of these miRNA change with disease progression and development of heart failure, but also exhibit changes as a function of age [147].

In conclusion, more studies are needed to examine the role of exosomes as mediators of these CVDs and further research towards these directions is needed to broaden the application range of exosomal biomarkers.

## 6. Concluding Observations

Research on the biology, pathophysiological function, and potential clinical application of exosomes has increased exponentially over the past years and they have emerged as potential biomarker reservoirs for several pathological processes. In this review, we decided to focus mainly on the potential application of exosomes as a diagnostic/prognostic tool in CVDs and, as consequence, we discuss the pros and cons of methodologies actually used in exosome research. Exosomes provide several advantages as a potential biomarker: (1) they are extremely specific and sensitive since they carry biological cargo that reflect the pathophysiological condition of the parental cells; (2) they can be isolated from easily obtainable biofluids such as blood and urine [148]; (3) the biomolecules transported by exosomes, compared to free circulating counterparts, have an increased half-life since the exosomal membrane protects them from degradation [149,150,151,152]. Nowadays, these aspects have been extensively investigated in several pathological contexts such as oncology [13,15] and neurodegenerative diseases [7,9], but less in CVDs. However, the studies here reported clearly demonstrate that exosomes, alone or added to the classical biomarkers, could serve to pronounce a more precise diagnosis/prognosis.

Despite the promising potential of exosomes both as therapeutic and diagnostic tool in CVDs, the challenge to their real clinical translation is represented by technical limitations. The isolation and characterization and they should be standardized and simplified to ensure their feasibility in daily clinical practice. Moreover, these techniques should be suitable to the small volumes that can be obtained from patients in the clinical context. Furthermore, the isolation of exosomes without contaminating plasma/serum proteins and lipoproteins is crucial for their downstream molecular analysis and reproducibility. Along with isolation/characterization methodologies, also nomenclature should be standardized. Despite the recent guidelines of the International Society for Extracellular Vesicles (ISEV) [17], a lot of studies currently fail in clearly defining the class of vesicles investigated and this lack of clarity slows down the progress in the field. The potential of exosomes as a biomarker in CVDs must be carried out in large cohorts of human patients and the vesicles must be rigorously identified to avoid misinterpretation of the results. Another weakness of current published studies on exosomes in CVDs is the lack of exosome quantification in patients and control subjects. This aspect is critical for the clear interpretation of the data since, without exosome count, exosomal components studied (miRNA, proteins) are not normalized to the amount of exosomes. In conclusion, exosomal biomarkers are still in the early discovery stage but the hope is that, in the near future, exosomes will be a powerful diagnostic marker to determine the progress of cardiovascular disease already at early stages.

## Figures and Tables

**Table 1:** Commonly used purification techniques.

Method	Principle	Examples of Application in Cardiovascular Field
Ultracentrifugation	Exosomes are purified on the basis of their size. Sequential centrifuge steps with increasing force (last step typically ~100,000× *g*) **Pros:** large sample capacity **Cons:** expensive equipment, time consuming, not suitable for small volume, potential mechanical damage due to high speed centrifugation	Cell culture [43,44,45] animal model [43]
Density gradient ultracentrifugation	Exosomes are separated according to their density in a discontinuous density gradient medium **Pros:** reduction of co-isolated contaminants compared to ultracentrifugation alone **Cons:** Time consuming, labor intensive, not suitable for small volume, potential mechanical damage due to high speed centrifugation	*n*/A ^1^
Ultrafiltration	Isolation of exosomes is based on size through filter membrane with defined size-exclusion limit or molecular weight cut-off **Pros:** Low equipment cost, fast procedure **Cons:** co-isolation of contaminants, potential deterioration induced by shear stress	Cell culture [46] Patient sample [47]
Size exclusion chromatography	Isolation method, based on size, that uses columns with stationary phase consisting of resin particles with known porous size **Pros:** good purity, shorter processing time compared to differential ultracentrifugation **Cons:** low vesicle yield, dilution of the sample	Cell culture [48] Patient sample [40,49,50]
Immunoaffinity capture	Exosomes isolation is based on interaction between exosomal antigens and specific antibodies **Pros:** isolation of specific exosome subpopulation, good purity **Cons:** high-cost antibodies, low yield, low capacity	Cell culture [51] Patient sample [52,53]
Polymer-based Precipitation	Exosome solubility is altered by water-excluding polymers **Pros:** easy to use, no specialized equipment, high recovery rate, suitable with small volumes **Cons:** Co-purification of contaminants, polymer may interfere with the downstream analyses	Cell culture [54,55] Patient sample [52,56,57,58,59,60,61]
Microfluidics-Based Techniques	Microscale isolation based on different principles including immunoaffinity, size and density **Pros:** Highly efficient, low cost **Cons:** not suitable for large volume, lack of method validation	*n*/A

^1^*n*/A not available.

**Table 2:** Commonly used characterization techniques.

Method	Description	Examples of Application in Cardiovascular Field
Nanoparticle Tracking Analysis (NTA)	**Parameter analyzed:** Dimension and concentration **Principle:** The particles in suspension are detected by incident laser light while a light-sensitive CCD camera records the scattered light of the particles and, finally, the software tracks the Brownian motion of vesicles and calculates their diameter based on the Stokes–Einstein relationship. **Pros:** Easy sample preparation, fast analysis/measurements, sample acquisition performed in a liquid phase, high resolution, vesicles are directly observed. **Cons:** Possible overlaying effect of larger vesicles, fail to distinguish exosomes from other nano-contaminants, determination of the proper dilution of the sample.	Cell culture [44,46,89] Patient sample [40,50,52,53,59,90]
Dynamic Light Scattering (DLS)	**Parameter analyzed:** size **Principle:** Monochromatic and coherent laser beam passes through a suspension of particles and the laser light fluctuations in scattered light intensity due to their Brownian movement is detected. **Pros:** high resolution **Cons:** Reliable data only when working with a monodispersed suspension, fail to distinguish exosomes from other nano-contaminants.	*n*/A ^1^
Tunable resistive pulse sensing (TRPS)	**Parameter analyzed:** Size, concentration, and surface charge **Principle:** Electrical resistance of the particles passing through a size tunable nanopore filled with electrolyte. Passing through the pore, the particle resistance increases, generating a pulse that is directly proportional to the particle volume. **Pros:** information about surface charge of vesicles **Cons:** Pores may be easily blocked by particles, particles may be too small to generate a signal higher than the background noise of the system.	*n*/A
Transmission Electron Microscopy (TEM)	**Parameter analyzed:** Morphology and size **Principle:** Images are generated from a beam of electrons passing through a sample generating secondary electrons, collected and magnified using special lenses. **Pros:** High resolution, discrimination between exosomes and other similar-size contaminants, immunostaining. **Cons:** change in the morphology of exosomes due to sample preparation, potential damage of sample by electron beam.	Cell culture [54] [46,51] Patient sample [47,59,60]
Atomic Force Microscopy (AFM)	**Parameter analyzed:** structure, biomechanics, and biomolecular content of individual exosomes. **Principle:** interactions between a probing tip and the sample surface. **Pros:** Minimal sample preparation and without any destructive mode of operation. **Cons:** analysis may be influenced by temperature, state of the tip, scan speed.	*n*/A
Flow Cytometry	**Parameter analyzed:** detection of exosomal marker **Principle:** new generation of flow cytometers use multiple angles for forward scatter detection to improve particle resolution. **Pros**: fast analysis **cCns**: Exosomes fall below the resolution of flow cytometry.	Patient sample [47,53,90]
Exoview platform	**Parameter analyzed:** cargo, size, concentration. **Principle:** Exosomes immobilized on ExoView chips by affinity capture and exosomal cargo can be studied, permeabilizing sample to enable access of antibodies. **Pros:** small volume, no vesicles isolation required, low purification biases. **Cons:** expensive instrumentation, time consuming.	*n*/A

^1^*n*/A not available.
